# The Impact of “Practice–Feedback–Journal” Microteaching Model on Critical Thinking Development in Chinese Pre-Service Teachers

**DOI:** 10.3390/bs15121745

**Published:** 2025-12-16

**Authors:** Zhiyao Tan, Qian Gong, Jia Liu, Feifei Liu, Liyun Lu, Wenxin Deng

**Affiliations:** School of Teacher Education, Nanjing Normal University, Nanjing 210023, China; 232012358@njnu.edu.cn (Z.T.); 242012308@njnu.edu.cn (J.L.); 69008@nnu.edu.cn (F.L.); luliyun@njnu.edu.cn (L.L.); 252012425@njnu.edu.cn (W.D.)

**Keywords:** critical thinking, microteaching model, qualitative analysis, pre-service teachers

## Abstract

Critical thinking, regarded as an indispensable high-order thinking for pre-service teachers, is of great significance in effective teaching and the cultivation of students’ critical thinking. Given that it has been confirmed by previous studies that the development of pre-service teachers’ critical thinking can be promoted by microteaching, a “Practice-Feedback-Journal” microteaching model was innovatively constructed in this study, aiming to cultivate the critical thinking of pre-service teachers. To investigate the impact of this model, 41 participants from a Chinese normal university were selected for a 12-week microteaching intervention experiment. Subsequently, content analysis of their online reflective journals was conducted using the Nvivo 20 software, based on the critical thinking framework. The findings suggest that the model has the potential to enhance Chinese pre-service teachers’ critical thinking. Evidence indicates that reflective feedback and case-based teaching strategies appear supportive, while cooperative learning also emerged as a promising approach due to its dual simulation of both student and teacher perspectives. As a small-scale exploratory study, this research offers preliminary insights and potential avenues for cultivating higher-order thinking in teacher education.

## 1. Introduction

The core value of higher education lies not merely in awarding diplomas to students, but more profoundly in shaping their ways of thinking (Lau, 2015). Critical thinking, as an essential competency for university graduates (Zou’bi, 2021), represents a form of higher-order thinking. It constitutes a key element of such thinking and encompasses both skill and dispositional dimensions (Zhong, 2020). Through the teaching of relevant skills, learners can acquire critical thinking abilities and thereby cultivate a critical mindset (Zhong, 2020). For teacher-training institutions in higher education, it is particularly important to emphasize the development of higher-order thinking with critical thinking at its core when preparing future educators (Zhou et al., 2023). This is crucial to ensure that they can meet the demands of the 21st century and enhance the quality of education (Astuti et al., 2019). As the cadre of the teaching profession, pre-service teachers must equip themselves with critical thinking skills to foster the same abilities in their students (Brandt et al., 2021). There was also a study showing a significant positive correlation between teaching effectiveness and teachers’ critical thinking skills (Birjandi & Bagherkazemi, 2010). Therefore, it is crucial for pre-service teachers to develop critical thinking for effective teaching and successful guidance in students’ thinking pattern construction.

Given the significance of critical thinking for pre-service teachers, numerous scholars have explored various effective teaching methods such as inquiry-based teaching (Verawati et al., 2019), creative drama (Uzunöz & Demirhan, 2017), and journaling (Kaplan et al., 2007) to promote the development of pre-service teachers’ critical thinking skills. However, some researchers have found that the current state of critical thinking among most undergraduate pre-service teachers was at a medium level or slightly below, and critical thinking was not effectively cultivated in Chinese teacher education by reviewing empirical research data (J. Bi & Che, 2024).

In China, microteaching has evolved into a relatively mature curriculum and training system in normal universities, which are specialized teacher-training institutions. Within this system, pre-service teachers participate in various simulated teaching activities such as instructional design, teaching practice, and discussions. They utilize various forms of feedback in microteaching to scrutinize and reflect on their teaching behaviors, thereby optimizing their teaching practices (L. Liu et al., 2023). Studies have confirmed that microteaching has a significant impact on the development of critical thinking among pre-service students (Arsal, 2015). However, existing research has highlighted many shortcomings in microteaching, such as an overreliance on video feedback and a lack of understanding regarding diverse feedback strategies (Huang & Wei, 2012). Considering that scrutiny and reflection processes are supported by feedback (L. Liu et al., 2023), and that reflection is a core element of critical thinking (Zhong, 2020), it follows that without a reasonable microteaching model, the cultivation of pre-service teachers’ teaching reflection abilities will be ineffective (Zhang, 2015), which in turn limits the development of their critical thinking.

This study, while ensuring the normal teaching progress of pre-service teachers, proposed an immersive microteaching model called “Practice–Feedback–Journal” (PFJ) to cultivate their critical thinking by improving the single feedback mechanism, strengthening the reflection process, and introducing journal writing to deepen reflection. The study uses Murphy’s (2004) critical thinking model as the analytical framework, which is focused on online environments and includes five processes: Recognize, Understand, Analyze, Evaluate, and Create. It can identify the dynamic changes in critical thinking skills, the overt manifestations of critical thinking, and is suitable for analyzing online-submitted reflective journals. This study applied Murphy’s critical thinking model to analyze the online-submitted reflective journals of pre-service teachers, exploring their behavioral manifestations and changes in each process of critical thinking skills after experiencing the PFJ model, thereby assessing the effectiveness of the model.

## 2. Literature Review

### 2.1. Definition and Value of Critical Thinking

Critical thinking, a concept with its root in ancient Greece, centers on making appropriate judgments based on specific criteria (Hou et al., 2022). This concept has been explored and developed in academic research, leading to a plurality of definitions. Dewey referred to critical thinking in *How We Think* as reflective thinking, a process of repeated, serious, and sustained reflection on an issue. Ennis (1962) defined critical thinking as the reasonable and mature thinking involved in determining what to believe or do. Watson and Glaser (1980) stated that critical thinking was both a skill and an attitude that encouraged individuals to interpret information from a more complex perspective, thereby making wiser decisions and solving complex problems in the real world (Butler et al., 2012). Halpern (1998) noted that critical thinking was not only an internal thinking disposition but also a set of skills or cognitive strategies that could be learned. Some scholars have also pointed out that critical thinking encompasses multiple aspects, including memory, comprehension, analysis, evaluation, reasoning, and reflective judgment (Dwyer et al., 2014). This multifaceted nature reflects the differing emphases among scholars regarding its definition. Some emphasize critical thinking as a skill for interpretation and evaluation (A. Fisher, 1997), others highlight analysis and evaluation (Paul & Elder, 2019). while others focus on reflection and reasoning (Ennis, 1985). Additionally, some view critical thinking as encompassing intellectual skills such as reflection, self-regulation, analysis, reasoning, interpretation, synthesis, and systematic thinking (Dumitru & Halpern, 2023). Despite the differences in the definition of critical thinking, there is a consensus on several basic points. Firstly, critical thinking encompasses not just reflection, but also the collection and extrapolation of evidence, and the reconstruction of reasonable judgments. Secondly, critical thinking should not be confined to the cognitive level but extend to the explicit skills one possesses. Thirdly, the key processes within critical thinking skills are recognition, understanding, analysis, evaluation, and creation.

There exists unique value for pre-service teachers in the domain of critical thinking. The Holmes Group (1986) of the United States emphasized that future teachers should not only have a deep understanding of their students, a systematic mastery of their subject knowledge, and a profound grasp of the nature of school education, but also serve as models for the development of critical thinking in their students. As future educators, the critical thinking of pre-service teachers directly influences the development of critical thinking in their future students (Yuan et al., 2022). Thus, the cultivation of critical thinking among pre-service teachers is key to improving the overall critical thinking skills of future adolescents (J. Bi & Che, 2024), which is important to prepare them for the post-industrial era or the era of Industrial Revolution 4.0 in the 21st century (Hussain, 2021). If pre-service teachers themselves lack sufficient experience in reflection and critical thinking yet attempt to teach these skills, they will inevitably fall into a logical contradiction (Bezanilla et al., 2021). Dong (2015) emphasized that critical thinking education, anchored in rationality and openness, was crucial for developing talents with creative abilities, which were necessary but underdeveloped in China. Critical thinking and creative thinking, as the core components of higher-order thinking, work in synergy and complement each other; critical thinking provides the logical foundation for creative thinking, while creative thinking is the continuation of critical thinking. Pre-service teachers are responsible for shaping future innovators, so the development of their own critical thinking becomes a key part of this process (J. Bi & Che, 2024). Therefore, it is important to develop an effective teaching program to improve critical thinking of pre-service teachers.

Critical thinking does not develop automatically; its cultivation relies on conscious and supportive educational interventions. Dewey placed reflective thinking at the core of experience and inquiry, emphasizing that reflective thinking must be systematically developed through guided “learning by doing,” thereby establishing the teachability of higher-order thinking (Dewey, 1910). From the perspective of professional practice epistemology, Schön argued that fostering the ability to “reflect in action” requires a learning environment that supports experimentation and framework reflection (Y. Li & Zhang, 2024). Lipman through the establishment of “communities of inquiry,” provided systematic curricular validation, demonstrating that higher-order thinking can be effectively taught within specific socio-cognitive environments (Lipman et al., 1980). Fisher’s research further translated theory into actionable classroom practices, illustrating how thinking can be transformed into assessable outcomes through explicit instructional design (R. Fisher, 2007). Brown contended that the foundation of teaching critical thinking lies in whether education acknowledges and safeguards learners’ right and freedom to “question and construct autonomously” (Brown, 2002) Thus, the development of critical thinking depends on multidimensional educational interventions encompassing philosophical foundations, practical environments, curricular methods, and the safeguarding of intellectual rights.

### 2.2. Definition and Value of Microteaching

Microteaching, as an instructional practice model implemented in controlled environments, centers on the core strategy of having teacher candidates engage in systematic activities such as instructional design, trial teaching, and follow-up discussions during simulated teaching sessions. In this process, candidates make full use of the diverse feedback mechanisms provided by the microteaching platform to closely examine and reflect on their own teaching behaviors, continuously refining their teaching competencies (Arsal, 2014; M. Liu, 2023). Contemporary microteaching emphasizes reflective practice and the development of teaching skills (Cavanaugh, 2022). Multiple studies, examining microteaching from the perspectives of both teacher trainers and preservice teachers, generally indicate that microteaching is an effective and practical tool for fostering self-reflection and ongoing professional development (Park, 2021). Currently, most normal universities in China adopt a technical imitation approach for microteaching, which involves breaking down teaching skills into individual components. After the explanation and demonstration of teachers, students practice in a targeted way, and then receive feedback and continue to practice (J. Li, 2019), which is the cyclical process of “teaching design-practice-feedback-redesign-practice” (J. Li, 2019). Commonly used feedback strategies include video self-reflection, peer feedback, and teacher feedback. Video self-reflection allows pre-service teachers to gain a more nuanced understanding of microteaching practice and engage in immersive reflection (Walshe & Driver, 2019). Peer feedback provides opportunities to observe and interact with peers, which can have a significant impact on pre-service teachers’ practice (Odo, 2023). And teacher feedback enables teachers to promptly raise issues and provide correct demonstrations during pre-service teachers’ trial lessons (M. Wang et al., 2025).

Existing research have suggested that microteaching provides multi-dimensional benefits for pre-service teachers, including assessing personal strengths and weaknesses (Ga et al., 2025), enhancing self-confidence (Walshe & Driver, 2019), constructing professional identity and identity roles (Farrell, 2011; Yuan & Mak, 2018), facilitating reflective thinking (Alamri & Alfayez, 2023) and enhancing critical thinking development (Danday, 2021). Therefore, combining critical thinking development and microteaching is a feasible and productive approach.

### 2.3. Development of Critical Thinking Based on Microteaching

Some studies have showed that there are still many deficiencies in the feedback session of microteaching, such as an overreliance on video feedback (Alamri & Alfayez, 2023) and a lack of understanding of multiple feedback strategies (Huang & Wei, 2012). Additionally, microteaching lacks a structured reflection session, with reflection activities frequently remaining superficial and failing to address the underlying issues (Huang & Wei, 2012; Karakas & Yükselir, 2025; Schön, 1987). If pre-service teachers merely imitate the teaching language, body movements, behaviors, and processes of exemplary teachers without engaging in proactive contemplation and reflection on the rationale behind these practices, it will be challenging to effectively combine theory with practice, to have a deeper comprehension of the implicit knowledge in teaching and to achieve advancement (J. Li, 2019). Moreover, they are even more unable to develop critical thinking centered on reflection (Zhong, 2020). Therefore, if microteaching lacks scientific teaching strategies, it will be difficult to fully realize its potential in developing pre-service teachers’ teaching reflection skills (Zhang, 2015), which in turn affects their development of critical thinking centered on reflection. This also embodies the core principles of Dewey’s “learning by doing” and Schön’s “reflection in action.” Therefore, microteaching, as a practice that simulates real teaching scenarios, provides teacher candidates with an immersive field for thinking training.

The teaching strategies for critical thinking proposed by Ennis (1989), including general, infusion, and immersion strategies, remain instructive today. General strategies promote students’ critical thinking skills by explicitly stating the basic principles of critical thinking and supplementing them with practical exercises (Cáceres et al., 2020). Studies such as Paul and Elder’s (2019) are precisely based on this concept to design a series of interventions aimed at developing students’ critical thinking. Infusion strategies involve teaching the principles and practical application of critical thinking more directly and explicitly and are achieved by integrating critical thinking into the teaching of other disciplines. For example, McLaughlin and McGill (2017) and others focused on the school history curriculum to develop students’ ability to identify pseudoscientific information. Immersion strategies do not place particular emphasis on explicitly articulating the rules of critical thinking but rather focus more on students’ experience and reflection in real-world contexts. For example, Huber and Kuncel (2016) noted that engaging students in the exchange of ideas and civic engagement in rigorous thinking activities could promote the development of critical thinking more effectively.

Integrating the cultivation of thinking skills with curriculum instruction involves selecting specific teaching methods, which are the tangible manifestations of various components within teaching models. Critical thinking teaching models are primarily manifested as three types: the direct teaching model of specialized training, the indirect teaching model based on realistic consideration, and the integrated teaching model of complex thinking (Chen, 2014). The direct teaching mode requires students to master the knowledge information provided by teachers and imitate it accurately (Costa, 1985), which is demanding and difficult to operate in terms of the design of teaching content and the preparation of teaching materials. The indirect teaching model, on the other hand, allows students to learn how to think through critical thinking through teacher guidance (Chen, 2014), and the specific methods include dialogue, free-questioning, debate, inquiry-based learning, self-assessment assignments, and reflective journal writing, all of which have been proven to be effective in enhancing students’ critical thinking skills (Almulla & Al-Rahmi, 2023; Fung, 2014; Golding, 2011; Lee et al., 2013; L. Liu et al., 2023; Qatipi, 2011; Siles González & Ruiz, 2012). The integrated teaching model, which combines direct and indirect teaching modes, integrates open-ended discussion strategies, inquiry strategies, value clarification strategies, and concept development strategies (Costa, 1985), is more difficult to operate.

Given that the participants would directly enter the internship phase after the microteaching and that course time was relatively tight, to ensure that the normal progress of curriculum instruction was not affected, the study employed the immersive critical thinking teaching strategy, emphasizing the integration of thinking skills with curriculum instruction. To leverage the advantages of different feedback strategies, this study draws on Lipman’s “community of inquiry” model and designs a diversified feedback approach including video self-reflection, peer feedback and teacher feedback. By establishing clear dialogue rules, discussions are guided to move beyond sharing initial impressions of trial teaching, to analyzing the educational rationales behind teaching behaviors and exploring their potential impact on students, thereby cultivating critical thinking through interaction. To prevent geography pre-service teachers from merely imitating exemplary teaching cases within their group or superficially accepting feedback without deeper reflection, this study incorporates reflective journals as tangible outputs to drive reflective practice during the training phase. The use of reflective journals traces back to Schön’s theory of reflective practice, where journal writing itself serves as a reflective dialogue with one’s own practice, helping pre-service teachers move beyond surface-level descriptions of teaching behaviors to critically examine the underlying assumptions and decision-making frameworks. The effectiveness of reflective journaling in stimulating critical thinking and developing metacognitive awareness has been widely recognized (Celik, 2014; Farrell & Kennedy, 2020). Through writing reflective journals, pre-service teachers contextualize their practice, thinking processes, and reflections, engage in self-assessment, and effectively enhance reflection, metacognition, and self-evaluation, thereby fostering critical thinking (Siles González & Ruiz, 2012). This study also made use of the current well-functioning Superstar Learning Platform, which made the collection of journals convenient. Based on the aforementioned teaching strategies, feedback strategies, journal-driven reflection methods, and platform support, this study proposed a microteaching model of “Practice–Feedback–Journal”, in which each pre-service teachers experienced peer-feedback, teacher-feedback, and self-feedback sessions after a trial lecture, and then wrote and submitted a reflective journal on the Superstar Learning Platform. There are three main research questions:

1. What Is the Current Situation of Critical Thinking Among Chinese Pre-Service Teachers?
2. Does the Pfj Microteaching Model Enhance Pre-Service Teachers’ Critical Thinking?
3. Are there variations in the effects of various teaching methods employed by pre-service teachers on their own critical thinking development?

## 3. Methodology

This study collected the microteaching reflection journals of pre-service teachers from a normal university in China, and adopted the content analysis method to analyze the reflection journals submitted online by using Murphy’s critical thinking model (see Appendix A), exploring their behavioral performances and changes in various processes of critical thinking skills they exhibited after experiencing the PFJ model, thus assessing the effectiveness of the PFJ model.

### 3.1. Participants

**Figure 1:**
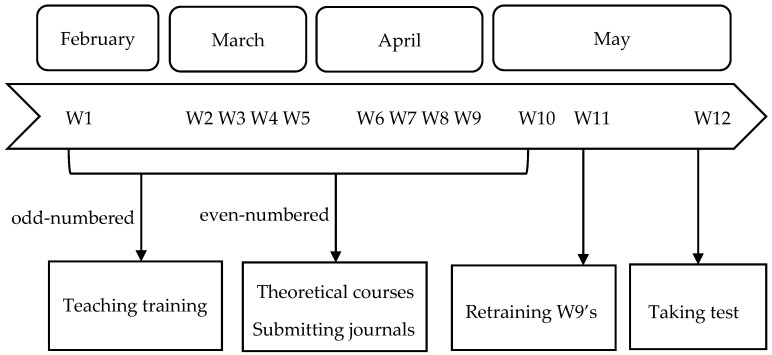
Experiment procedure.

The participants of the study were 41 pre-service teachers in their third undergraduate year at a Chinese normal university. They had completed core teacher-education courses, including pedagogy, educational psychology, modern educational technology, and curriculum and instruction, thus possessing a foundational understanding of educational theory and practical teaching skills. All participants joined the project on a voluntary basis, with no conflicts of interest. The study spanned 12 weeks and was conducted through a combination of theoretical explanations by the instructor and microteaching by the pre-service teachers. Microteaching was a comprehensive process that included microteaching practice, multi-feedback, and reflective journal writing. That is, after each practice session, the participants would experience peer feedback, teacher feedback, and self-feedback, and then would be required to write and submit reflective journals (Figure 1). A total of six microteaching sessions were conducted in the experiment, and teaching reflection journals were submitted five times (the sixth microteaching session was a redesign of the fifth one, and no teaching reflection journals were submitted). The content covered five different teaching themes: “The Heating Process of Atmosphere”, “Water Cycle”, “Urbanization”, “Geographical Significance of Earth Movement”, “Pressure Zones and Wind Zones”, four teaching methods: the lecture method, the cooperative learning method, the demonstration method and the board drawing method, as well as one teaching plan presentation.

### 3.2. Data Collection and Analysis

This study draws on data from 41 geography pre-service teachers who submitted five rounds of teaching reflection journals via the Superstar Learning Platform. To facilitate tracking and analysis, the raw data were first documented and organized by creating individual files for each student according to teaching topics. These files were uniformly named using the student ID followed by the student’s name to enable systematic analysis of critical thinking performance. Building on this, the study established a theoretical framework based on Murphy’s critical thinking model, clarifying the dimensions of critical thinking skills to be assessed. This model deconstructs critical thinking into five cognitive stages: recognition, understanding, analysis, evaluation, and creation, each corresponding to a set of observable and assessable behavioral indicators.

During the data analysis phase, open coding was conducted using Nvivo 20 based on Murphy’s critical thinking model. Tree nodes were assigned to represent the five stages, while child nodes were used to denote specific behavioral performances under each stage. Specifically, content in the journals that reflected critical thinking was categorized into the corresponding behavioral indicators of the model (Table 1). Given that a single text may involve multiple stages or behaviors, a multi-node parallel coding strategy was adopted. Furthermore, to eliminate bias caused by variations in text length, the analysis did not rely on absolute frequencies. Instead, the node proportion was used as a comparable indicator to reflect the relative intensity of critical thinking performance across stages. For example, the total number of nodes for the “understanding” stage (U) was calculated by summing the frequencies of all its child nodes (U1 to U6), and then dividing this sum by the total number of nodes in the journal.

## 4. Results

### 4.1. Descriptive Analysis of Critical Thinking Level of Pre-Service Teachers

**Figure 2:**
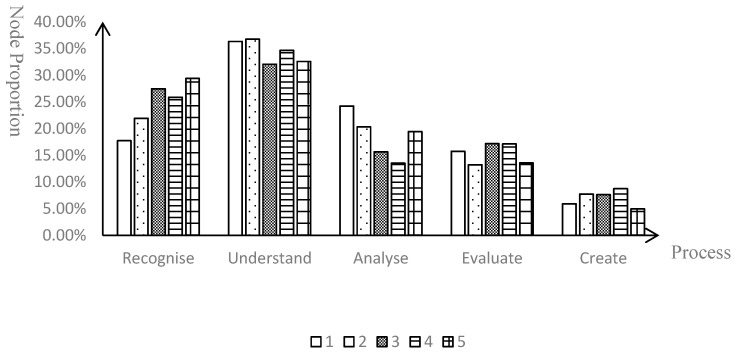
Node proportion in each process of critical thinking.

The critical thinking levels of pre-service teachers were relatively low, yet the vast majority possessed the ability to recognize. To clarify the performance of these pre-service teachers in various processes of critical thinking, the study conducted coding on the 5 teaching reflection journals submitted by each of the 41 pre-service teachers and statistically analyzed the proportion of tree nodes at each process based on the coding results. Figure 2 indicates that the critical thinking of pre-service teachers was mainly concentrated on the understanding process, where they demonstrated a deep understanding of the teaching content and their own performance. In comparison, the proportions for the creation and evaluation processes were relatively low, suggesting that there was significant room for improvement in these areas for pre-service teachers. Furthermore, the study counted the number of documents involved in each process, with the number of documents representing that of pre-service teachers (Table 2). This reveals that while the majority of pre-teachers had the ability to discern and identify issues within the microteaching, only a minority of them possessed the ability to create.

### 4.2. Effectiveness of the Pfj Model in Different Processes and Behaviors

**Figure 3:**
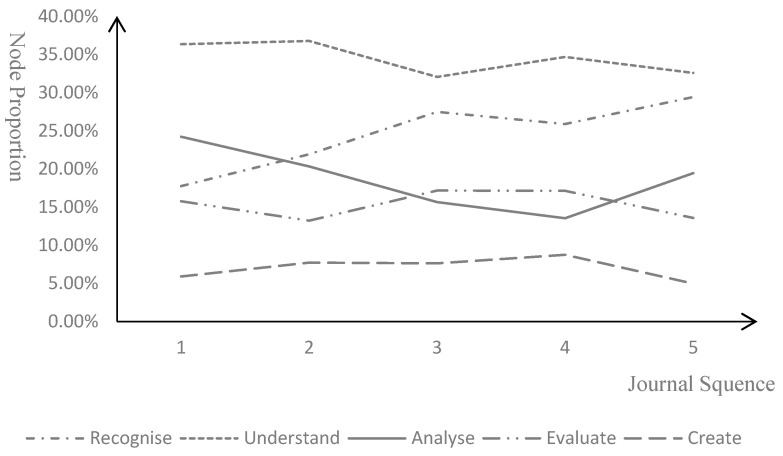
Node-Proportion Changes in Each Process of Critical Thinking.

The PFJ model showed varying degrees of effectiveness in cultivating the critical thinking in different processes and specific behaviors among pre-service teachers. To explore the model’s impact on the development of critical thinking in different processes, a statistical analysis was conducted on the proportion of tree nodes at each process within five reflective journals (Figure 3). The results indicated that in the understanding and creation processes, the change of the proportion of tree nodes remained relatively stable, suggesting that the PFJ model’s effectiveness in enhancing critical thinking at these processes was not significantly pronounced. In the recognition process, there was a notable increase in the proportion of tree nodes, indicating that the PFJ model enabled pre-service teachers to more keenly recognize key issues and challenges in teaching. The proportion of tree nodes in the analysis process showed a decline, which suggested that more emphasis should be placed on training pre-service teachers’ analytical skills subsequently. It was also indicated that the proportion of tree nodes fluctuated in the evaluation process, which not only reflected the pre-service teachers’ unstable evaluative capabilities but also implied the need to further optimize the model to strengthen their performance in the evaluation phase.

**Figure 4:**
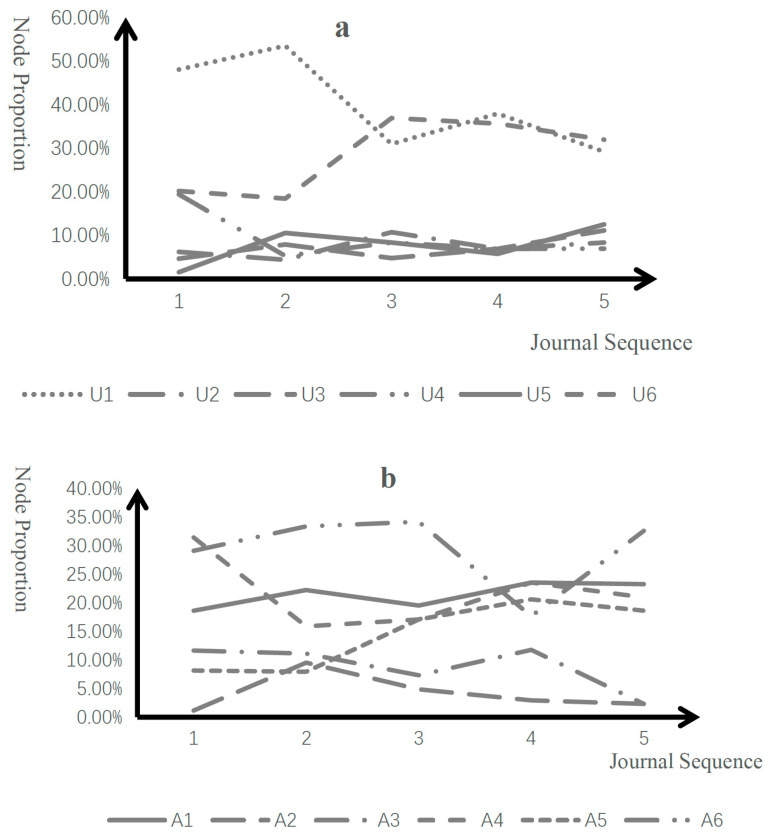

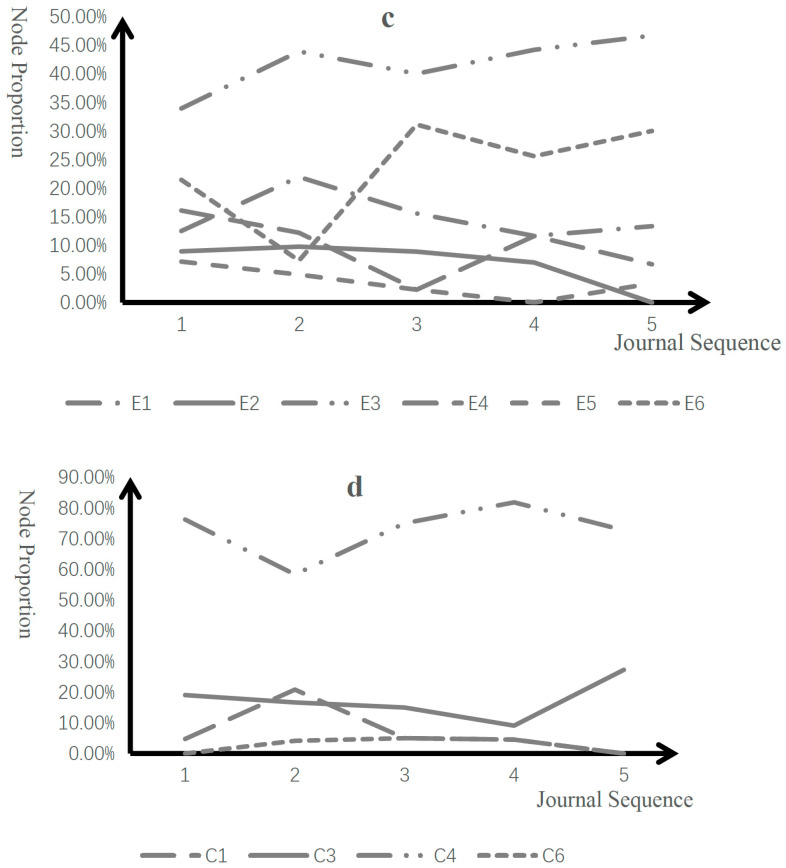
Node-Proportion Variations of Specific Behaviors in Critical Thinking Processes Note: C2 and C5 were absent. (**a**) Node proportions in the understanding process; (**b**) Node proportions in the analysis process; (**c**) Node proportions in the evaluation process; (**d**) Node proportions in the creation process.

To assess the effectiveness of the PFJ microteaching model on the cultivation of specific critical thinking behaviors among pre-service teachers, a statistical analysis of the sub-node proportions at each process within the five reflective journals was conducted (Table 3, Figure 4). In the recognition process (Table 3), the proportion of R1 significantly increased, which indicated that the PFJ model markedly enhanced the pre-service teachers’ ability to identify issues, thus laying the foundation for subsequent in-depth analysis and resolution of the problems. As for the understanding process (Figure 4a), the proportion of U6 significantly increased, suggesting that the PFJ model effectively stimulated active thinking and interaction among geography pre-service teachers, which was positively significant for their knowledge internalization and cognitive expansion. The proportion of U1 fluctuated and decreased, yet it still occupied a substantial proportion in the understanding process, indicating that fundamental information perception and understanding play a crucial role in the cognitive development of pre-service teachers. The variation in proportion can be interpreted as an adaptive allocation of internal resources within the understanding process, rather than a degradation of capabilities.

In the analysis process (Figure 4b), the proportion of A5 significantly increased, indicating that the PFJ model significantly enhanced the pre-service teachers’ ability to deconstruct complex problems, thus constructing a cognitive ladder for further in-depth analysis of teaching issues. In the evaluation process (Figure 4c), E1 dominated and showed an upward trend, while E5’s proportion fluctuated and rose to become next to E1. It indicated that this model encouraged pre-service teachers to learn to use evidence to judge the effectiveness, scientific validity, and relevance of teaching content, which was one of the goals of microteaching and significantly enhanced the teaching professional literacy and practical capabilities of pre-service teachers. The relative decline in the proportions of other evaluation behaviors was also intrinsically linked to the rise in the proportions of E1 and E5, reflecting the developmental path changes in the evaluation behaviors of pre-service teachers.

In the creation process (Figure 4d), C4 accounted for the main proportion and showed an upward trend, indicating that this model had a positive impact on the cultivation of innovative thinking. C6, C3, and C1 show little change. Based on cognitive psychology, self-cognitive schemas greatly influences the classroom behaviors and cognitive processes of pre-service teachers, suggesting that the development of these capabilities requires a long cycle or stimulation under specific conditions. C2 and C5 were absent, revealing a significant lack of ability among pre-service teachers to implement new plans. It suggested that future instructional strategies should focus on and strengthen targeted training for weak links.

### 4.3. Impact of Teaching Method Selection on Critical Thinking Development

The Cooperative Learning method stood out in comparison to other teaching methods in significantly influencing the cultivation of specific critical thinking behaviors among pre-service teachers (Figure 4). This study guided pre-service teachers in designing and practicing 4 different teaching methods: lecture method, the cooperative learning method, demonstration method, and blackboard drawing method. Based on the statistical results of sub-nodes, it then compared the specific behavioral performance in the four teaching methods. The findings indicated that the cooperative learning method designed by pre-service teachers during the third microteaching had an impact on their critical thinking. This method centers around clear tasks, group formation, collaborative learning, outcome presentation, reflection and evaluation, requiring students to discover and remedy deficiencies in knowledge or information through communication, and to engage in mutual judgement through interactions among peers and between teachers and students. Data analysis revealed that the cooperative learning method led to a significant increase in R1 behavior and an increase in U6, A6, and E6 behaviors, while U1 and E4 behaviors decreased. This is attributed to the high alignment of the cooperative learning method’s components with critical thinking behaviors R1, U6, A6, and E6, which emphasizes in-depth exploration, analysis, and evaluation of information within interactive communication contexts. In contrast, U1 behavior, based on initial understanding of established information, and E4 behavior, involving the formulation and judgement of definitions, are more closed-ended and do not align with the learning environment and thinking patterns fostered by the cooperative learning method.

## 5. Discussion

### 5.1. Current Situation and Constraints of Critical Thinking Development Among Chinese Pre-Service Teachers

Although pre-service teachers underwent the PFJ microteaching intervention, the findings suggest that their critical thinking development may still be limited. This finding corroborates with previous findings by X. Li and Liu (2021), highlighting the issues within the Chinese educational environment, which is predominantly exam-oriented. In this environment, understanding and recognition skills are reinforced due to their direct link with examination demands, while more advanced critical thinking skills such as creation and evaluation lack exam-oriented incentives, leading to a deficiency in student performance in these activities. Therefore, educators should focus on transcending mere problem identification in the cultivation process of pre-service teachers, guiding them to delve into thorough evaluation and effective problem resolution to foster the development of higher-order critical thinking.

The current situation may also be linked to the identity of pre-service teachers. Pre-service teachers, who are about to enter the teaching profession, place much emphasis on norms. During their training, they are encouraged to emulate teaching methods, which involves understanding the instructional designs of exemplary teachers. In this process, pre-service teachers may easily adopt outstanding teaching designs directly, which will lead to a direct thinking pattern and is not conducive to the formation of critical thinking. As Ma et al. (2015) pointed out, understanding a teacher’s logic is equivalent to grasping the knowledge conveyed by the teacher, and this approach to teaching can be detrimental to the cultivation of critical thinking among pre-service teachers. The prevailing education system, which prioritizes knowledge dissemination, also leads to the challenge of resource scarcity for nurturing critical thinking. Pre-service teachers are accustomed to lecture-based instruction and rote learning of standard answers. Over time, they may gradually lose the initiative for independent thinking and develop a passivity in receiving knowledge, thereby lacking critical thinking abilities (Ma et al., 2015). Constrained by traditional Chinese culture, especially “Honor Teachers and Respect Rules” emphasized by Confucian, parents and educators also influence pre-service teachers with this ideology, which may lead to their increasing compliance with authority and uniformity in thought, and to a certain extent, suppresses their questioning and critical spirit towards authoritative views, thereby impeding the development of critical thinking (Turner, 2006; Ziliotti, 2022).

### 5.2. Reflective Practice with Multi-Feedback and Case Teaching Enhancing Critical Thinking Development in Preservice Teachers

Firstly, the PFJ microteaching model appears conducive to the development of critical thinking among pre-service teachers and is particularly effective in nurturing recognition. The essence of PFJ is reflective teaching based on critical thinking. Reflection, which inherently involves truth-seeking, self-examination, and questioning, serves as a key driver in the development of critical thinking (Quinn et al., 2020). Reflective teaching emphasizes that teaching subjects engages in action research to continuously explore and address issues related to themselves, teaching objectives, and teaching tools (Xiong, 2002). And the core component of it is to focus on cultivating pre-service teachers’ ability to identify problems and difficulties clearly. Thus, this model may help facilitate pre-service teachers in recognizing issues within the teaching process, thereby developing their recognition skills.

Secondly, the multi-feedback mechanism seems to play a positive role in the understanding process of critical thinking among pre-service teachers. This mechanism is a collaborative learning model that includes collaboration among students and between teachers and students. During this collaborative process, the more interaction and discussion occur between teachers and students on a given topic, where critical thinking is authentically applied and developed, the more likely it is that the learning process of higher-order thinking will take place (Fernandes et al., 2024). X. Li et al. (2021) also believed that when learners were required to explain their viewpoints to others, it promoted the production of clear, structured thinking outcomes. This collaborative construction of knowledge is an effective way to develop higher-order thinking. Existing research has suggested that through the learning process involving peer feedback and self-feedback, pre-service teachers can engage in in-depth questioning and discussion on the rationality of arguments and demonstrate a strong inclination towards critical thinking, effectively promoting the development of critical thinking (Zou et al., 2023).

Thirdly, case teaching may contribute to improvement of pre-service teachers’ analytical skills. PFJ was conducted in groups, with each pre-service teacher serving as a case. After the trial lecture, each pre-service teacher was required to analyze the lecturer from concept to practical operation, engage in discussions, and form repeated interactions and exchanges. This case-based approach, grounded in peers’ authentic teaching practice, has been shown to markedly improve the analytical skills of pre-service geography teachers. This aligns with the findings of Bi on embedded case-based teaching for critical thinking development (X. Bi, 2023). The method effectively utilizes diverse learning resources by shifting from traditional instructional approaches to case-based learning. Through activities such as case analysis, problem discussion, sharing of learning outcomes, and teaching reflection—encompassing analytical evaluation, interpretation and explanation, reflection, and self-calibration—students’ abilities in problem analysis and resolution are enhanced (Dumitru & Halpern, 2023), thereby promoting the development of critical thinking.

Fourthly, feedback and reflection may significantly improve the performance of pre-service teachers in the evaluation process of critical thinking. In this model, pre-service teachers, based on multi-feedback and self-reflection, considered the content learned from different perspectives and judged whether their viewpoints and arguments were supported by evidence. This kind of thinking reflects critical thinking, which corroborates the research findings of Zou et al. (2023) that feedback and reflection are important for the development of critical thinking among pre-service teachers. At the same time, pre-service teachers learned to articulate knowledge or express viewpoints based on curriculum standards during the feedback and reflection process. This process involves a profound understanding of the nature of problems (Ikuenobe, 2001). For Chinese pre-service teachers who are accustomed to accepting authority, this method can break thinking sets, cultivate independent thinking and questioning abilities, thus becoming effective to enhance critical thinking (S. Wang & Seepho, 2017).

### 5.3. The Cooperative Learning Method Enhancing Critical Thinking Development in Teaching Subjects and Objects

The findings also indicate that the design and implementation of the cooperative learning methods may influence pre-service teachers’ critical thinking. Under the PFJ model, pre-service teachers hold dual roles, acting as both the teaching subjects (teachers), and the teaching objects (simulated students). For teaching objects (simulated students), the interactive process of the cooperative learning method is crucial for strengthening their critical thinking. During interactions, the collision of different viewpoints will inevitably lead to conflicts and contradictions. At this point, individuals are expected to reflect on the rationality of their own viewpoints, thereby stimulating critical thinking. This finding aligns with that of Smith et al.’s (2018). Almulla also confirmed through structural equation modeling that peer interaction has a direct and positive impact on critical thinking (Almulla & Al-Rahmi, 2023). Furthermore, the group collaboration method encourages students to actively identify and address gaps in their knowledge or information during exchanges, while also promoting reflection through mutual evaluation and feedback. By comparing their own performance with that of others, learners naturally advance the development of critical thinking (Lin et al., 2021). As Ikuenobe noted, group discussion is a process of analyzing, evaluating, and synthesizing multiple perspectives, offering participants opportunities to deeply examine their own views and respond to others’ (Ikuenobe, 2002), thus positively influencing the development of critical thinking (Lin et al., 2021).

In addition to the conclusion that cooperative learning method may enhance learners’ critical thinking (S. Wang & Seepho, 2017), this study further suggests this method’s potential positive effect on the critical thinking of the teaching subjects (teachers). When teachers employ this method in their instructional design, they are creating a platform conducive to critical thinking for their students. In practice, teacher–student interaction is a key component, which in itself is an exercise and enhancement of the teachers’ own critical thinking. As Snyder and Snyder (2008) pointed out, teachers who use the cooperative learning method should possess critical thinking themselves, set tasks that help developing it through activities, and pose open-ended questions to encourage free thinking. Therefore, in the design of teaching and microteaching practice, it may be advisable for pre-service teachers to prioritize open, cooperative teaching methods to foster the joint growth of critical thinking among both teachers and students, as well as ensure teaching effectiveness.

## 6. Conclusions

This study suggests that within the exam-oriented educational environment in China, pre-service teachers may have significant room for improvement in critical thinking due to the influence of their identity as pre-service teachers and the relatively limited resources available for the cultivation of critical thinking. The findings indicate that PFJ microteaching model, which is reflective at its core and incorporates multi-feedback and case teaching, appears beneficial for pre-service teachers to recognize, understand, analyze, and make evidence-based evaluations of issues, showing promise for fostering critical thinking. The selection and use of open, cooperative teaching methods in the process of instructional design and implementation may further help pre-service teachers promote their own critical thinking development.

This study has certain limitations. Firstly, the research sample cannot represent the situation of all pre-service teachers in Chinese universities, which covers only geography pre-service teachers from one normal university. Future research are expected to expand the sample size, considering the diversity of regions, school types, and other factors to enhance the universality of the research findings. Secondly, the changes in critical thinking are highly complex, as the changes are not influenced by a single factor, such as the teaching methods in this study. Therefore, it is clearly incomprehensive to simply use the number of course offerings as a measuring standard. Future research may strictly control relevant variables and adopt more scientific and systematic assessment methods.

The implementation of the PFJ model also presents several practical challenges. Firstly, the model requires balancing skill training, deep reflection, and multi-feedback within the limited time frame of a microteaching session, making equilibrium among these three aspects an ongoing design difficulty. Secondly, quality control of the multi-feedback mechanism poses a significant challenge. Peer feedback tends to be moderated out of consideration for interpersonal harmony, while self-reflection may remain superficial. Ensuring that feedback focuses on evidence and reasoning rather than personal preferences demands strong modeling and intervention from the guiding teacher, which places high demands on the guide’s own critical thinking and ability to facilitate classroom dialogue. Furthermore, participants simultaneously take on the dual roles of “teacher” and “simulated student.” How to support their perspective-taking and effective learning through instructional design is another aspect that requires careful handling during implementation. Therefore, future research and practice should not stop at model validation but should focus more on how to provide systematic training for guides, develop tools to support feedback quality management, and design more flexible course structures, thereby advancing the cultivation of critical thinking from a theoretical framework toward sustainable educational practice.

## Figures and Tables

**Table 1:** Coding example.

Code	Content Excerpt
R1	*I realized that before preparing lessons, I should conduct a good analysis of students’ learning situations, and …*(10210243 Qin)
U1	*I originally designed a lesson plan that was supposed to last within 10 min…I found that it actually took nearly 15 min…I think there are multiple reasons for this…*(10210239 Yuan)
U2	*In terms of the teaching content, in light of the curriculum standard “2.4 Use materials to illustrate the processes and characteristics of urbanization in different regions, as well as the advantages and disadvantages of urbanization”, the whole lesson lacked specific case analyses…*(10210119 Xu)
U3	*Due to self-designed lessons, there were a lack of some ingenious highlights in the settings of sections like experiments. In the future, it would be …*(10210446 Xu)
U4	*After listening to the evaluations from my teacher and fellow group members, I repeatedly replayed and carefully observed the problems in my teaching process, and thus gained new understandings and reflection.*(10210207 Wang)
U5	*… did not fully consider the characteristic of being close to real life in geography learning. It would be better to change it…*.(10210450 Chai)
U6	*Many classmates in our group made more vivid PowerPoint presentations than I did…I also asked the group members about the ways …*(10210227 Li)
A1	*For example, in this microteaching, when time is tight, we can directly transit…omitting the process… In the situation setting…For those less important links, they can be deleted…*(10210609 Liu)
A2	*The audience for a lesson presentation is teachers, while the audience for teaching is students…* (10210101 Ding)
A3	*Physical geography is more inclined towards established principles. Clearly explaining those principles to students can help them understand. However, human geography requires the combination of more practical phenomena and materials for specific analysis to obtain summary knowledge from the phenomena, which places higher demands on students’ comprehension level.*(10210118 Tang)
A4	*This time, in order to…an extracurricular material about “German industrialization” was added. However, there is still room for..*.(10210227 Li)
A5	*…three inquiry activities were mainly set up … the three main questions of “what”, “why”, and “how”, to help …*(10210352 Jing)
A6	*The key and difficult points of teaching should be…My entire teaching design only covered …*(10210213 Liu)
E1	*Although the introduction was designed to be relevant to geographical life… Some students may not be familiar enough with the geographical environment…which might affect their understanding …*(10210232 Zhou)
E2	*Ignoring the differences in the processes… I myself don’t understand the knowledge points…I only have a superficial knowledge and lack the ability to judge …*(10210104 Fang)
E3	*Relying solely on this experiment can only prove…It is impossible to rule out…lacking rigor in logic…A comprehensive consideration should be taken during the demonstration.* (10210207 Wang)
E4	*This “conflict” questioning method can make students have a deeper impression of this content*.(10210402 Wang)
E5	*The overall time control of the lesson presentation is relatively good, but there is still room … The stipulated time for this lesson presentation is 15 min, but it took me about 16 min…*(10210402 Wang)
E6	*The introduction of the course needs to be closely related to…This time I made a mistake…which was inappropriate.*(10210243 Qin)
C1	*In order to avoid…the previous micro-lesson…I conducted … also asked my classmates to act as students and carried out rehearsals of the group cooperation method…I arrived at the classroom in advance to…*(10210254 Cai)
C3	*In actual teaching, I personally think that it is possible to…the concept of…can also be* interspersed, *allowing students to…*(10210413 Liu)
C4	*During the correction process, it was found that students had a problem… these two knowledge points should be restated in front of the whole class, instead of… In this way, it can help…*(10210352 Jing)
C6	*This lesson has improved the problem …*(10210321 Zhang)

**Table 2:** Number of documents involved in each process of critical thinking.

	1	2	3	4	5
Recognize	41	41	40	40	39
Understand	33	37	30	36	33
Analyze	26	34	28	21	22
Evaluate	29	29	27	27	23
Create	15	18	19	20	9

**Table 3:** Changes in the proportions of sub-node in the Recognition process.

	1	2	3	4	5
R1	17.75%	21.94%	27.48%	25.90%	29.41%

## Data Availability

Requests for data can be sent to the corresponding author.

## Appendix A

**Table A1:** Critical Thinking Analysis Model.

Process (Tree Node)	Descriptor	Code (Sub-Node)	Specific Indicators
Recognize	Recognizing or identifying an existent issue, dilemma, problem, etc.	R1	Recognizing, identifying, or focusing on an issue, dilemma, problem, inner discomfort, or perplexity requiring further investigation or clarification.
Understand	Exploring related evidence, knowledge, research, information, and perspectives.	U1	Exploring and identifying what is relevant to the issue, dilemma, problem, etc.
		U2	Locating background information, knowledge, previously accepted conclusions, or evidence from other sources.
		U3	Locating alternate perspectives or evidence on the issue, dilemma, problem, etc.
		U4	Making observations.
		U5	Clarifying or appraising the nature of the issue, dilemma, problem, etc.
		U6	Questioning and exchanging information.
Analyze	Seeking in depth clarification, organizing known information, identifying unknown information, and dissecting the issue, dilemma, or problem into its fundamental components.	A1	Engaging in new ways of thinking and behaving.
		A2	Categorizing and classifying evidence, information, knowledge, or perspectives.
		A3	Differentiating similarities and differences in alternate perspectives or evidence on the issue, dilemma, problem, etc.
		A4	Interpreting and explaining the issue, dilemma, problem, etc.
		A5	Breaking down the problem, dilemma, issue, etc., into constituent parts.
		A6	Identifying and filling gaps in knowledge or information and judging one’s own thinking.
Evaluate	Critiquing and judging information, knowledge, or perspectives.	E1	Judging the validity, value, applicability, and relevance of information, knowledge, sources.
		E2	Critiquing perspectives and assumptions.
		E3	Detecting inconsistencies, fallacies, as well as correspondences and congruencies.
		E4	Making and judging definitions.
		E5	Using evidence to support arguments.
		E6	Retaining or rejecting evidence, information, knowledge, or perspectives.
Create	Producing new knowledge, perspectives, or strategies, and implementing them or acting on them.	C1	Implementing or executing strategies.
		C2	Applying actual or hypothetical solutions, decisions, or conclusions.
		C3	Constructing, creating, inventing, and devising new knowledge or perspectives.
		C4	Generating alternative hypotheses and perspectives.
		C5	Acting on a solution, decision, or conclusion.
		C6	Executing, or implementing change or a plan.
